# Inflammatory cell expression of Toll-like receptor-2 (TLR2) within refractory periapical granuloma.

**DOI:** 10.12688/f1000research.16678.1

**Published:** 2018-11-20

**Authors:** Eric Chen, Mahmoud M. Bakr, Norman Firth, Robert M. Love

**Affiliations:** 1 School of Dentistry, University of Otago, North Dunedin, Dunedin, 9016, New Zealand; 2 School of Dentistry and Oral Health, Griffith University, Southport, Queensland, 4215, Australia; 3 School of Dentistry, The University of Queensland, Herston, Queensland, 4006, Australia

**Keywords:** Antigen presenting cells, chronic inflammatory cells, immune response, periapical granuloma, toll-like receptors.

## Abstract

**Background:** Toll-like receptor-2 (TLR2) is highly important within the immune system. Characterization of the expression of TLR2 within inflammatory cells in periapical lesions could help in diagnosis and management of refractory cases. The aim of the study is identification of Toll-like receptor (TLR2) through immunohistochemical and immunofluroscence expression in inflammatory cells within refractory periapical granuloma cases.

**Methods:** Eight cases of refractory periapical granuloma were selected out of 772 cases. Histological examination and immunohistochemical staining with polyclonal rabbit antihuman TLR2, monoclonal mouse antihuman CD38, CD68 and CD83 primary antibodies, as well as immunofluorescence staining with goat anti-rabbit TLR2, donkey anti-mouse CD38, CD68 and CD83 primary antibodies was conducted. Positive controls, negative controls and experimental sections with no primary antibody were included in the study. Qualitative analysis and double immunofluorescence technique was used to characterize the TLR ^+ ^cells.

**Results:** In periapical granuloma, lymphocytes (CD38 cells) expressed the most amount of TLR reactivity followed by macrophages (CD68 cells), and odontogenic epithelial cells. Neutrophils, red blood cells (RBCs) and collagen ground substance were negative to TLR2.

**Conclusion: **TLR2 was highly expressed by lymphocytes and plasma cells indicative of their major role in the inflammatory process and antigen recognition in refractory periapical granuloma. Dendritic cells expressing TLR2 were low in number suggesting a minor role in sustaining these lesions.

## Introduction

The goal of conventional root canal treatment is to eliminate root canal microbes, or to substantially reduce microbial load and prevent re-infection by root canal filling and coronal seal. When this is achieved, healing of the peri-radicular tissue is expected. The environment of a well-treated root canal is harsh for most microbes as there are virtually no nutrients for growth. However, microbes associated with persistent intra-radicular infection take on this local ecological change and are viewed as opportunistic pathogens where microbial competitors are eliminated ^1^. However, residual microbes in the apical portion of the root canal system are the major cause of apical periodontitis persisting post-treatment in both poorly and properly treated cases ^2^. 

In refractory periapical lesions, pathogen recognition through innate immunity is achieved by several germline encoded pattern recognition receptors (PRRs). These receptors recognise pathogen-associated molecular patterns (PAMPs) that are conserved, unchangeable and essential for the survival or pathogenicity of the microorganisms ^3^. Some examples of PAMPs include lipopolysaccharides (LPS) of Gram-negative bacteria, Lipoteichoic acids (LTA) of Gram-positive bacteria, unmethylated CpG motifs of bacterial DNA, double-stranded RNA of RNA viruses and mannans of yeast cell walls. PRRs exist in several forms and are found in various locations. They can be expressed on the cell surface as transmembrane proteins (Lectin receptors and Toll-like receptors TLRs), in the intracellular compartments as enzyme (protein kinase PKR) or proteins (NOD-like receptors) or secreted into the bloodstream and tissue fluids as serum proteins (Mannan-binding lectin). The principle functions of PRRs include opsonisation and phagocytosis of pathogens, activation of complements and coagulation cascades, initiation of pro-inflammatory signalling pathways and induction of apoptosis ^4^. 

Toll-like receptors (TLRs) are type I integral membrane proteins on antigen presenting cells (APCs). Without an effective TLR system, the host is prone to microbial infection and subsequent fatality. It has an extracellular domain, a trans-membrane region and an intracellular domain. In brief, the extracellular domain recognises a variety of antigens during initial microbial challenge. The intracellular domain, with the help of other intracellular proteins, signals the activation of transcription factor NF-κB (nuclear factor kappa-light-chain-enhancer of activated B cells) and ultimately produces inflammatory cytokines that are responsible for mounting a more effective innate response and initiating adaptive immunity ^5^. Activation of NF-κB has been described as the master-switch of the immune system ^6^. 

Expression of TLRs is modulated by a variety of factors such as microbial invasion, microbial components and cytokines. Colony-stimulating factor 1 ^7^, macrophage immigration inhibitory factor ^8^, interferon (INF) -γ ^9^, IL-2, IL-15, IL-1β and TNF-α ^10^ are examples of cytokines that prime phagocyte response to microbial stimulation, up- or down- regulate TLR gene expression and augment inflammatory cytokine production. Zarember and Godowski ^11^ showed that most tissues express at least one TLR, and phagocytes/macrophages in particular show abundant expression of all known TLRs. Moreover, TLR3 is preferentially expressed in T helper cells (Th) and cytotoxic T cells (CTL), whereas B cells show a higher expression of TLR9, TLR 10. Mast cells have been shown to express TLR2, 4, 6 and 8 but not 5 ^12^.

TLR2 recognises components from a variety of microorganisms. Components that have been demonstrated are peptidoglycan and lipoteichoic acid (LTA) from Gram-positive bacteria ^13^, lipoprotein from Gram-negative bacteria ^14^, glycoinositolphospholipids from protozoa ^15^ and zymosan from fungi ^16^. Furthermore, TLR2 modulates the immune response through mediating the secretion of some antimicrobial peptides in response to bacterial lipoprotein, thereby regulating the direct killing of microbes at different parts of the human body ^17^.

In the pulp and peri-radicular tissues, TLR2 recognises peptidoglycan and LTAs that are major cell wall constituents of Gram-negative bacteria ^18^. *In vivo* immunohistochemical analysis has shown that TLR2 is expressed in the pulps of mice ^19, 20^ and human teeth ^21^. Interestingly, when stimulated with LTA *in vitro*, odontoblasts were able to upregulate their expression of TLR2 and initiate an innate immune response, secreting chemokines to recruit immature defensive cells (DCs) while down-regulating dentine matrix synthesis and mineralisation ^22^. The expression of TLR2 on odontoblasts reiterates their role as one of the major surveillance cells in the dentine-pulp complex. Furthermore, immunohistochemistry investigations revealed TLR2 expression in human refractory periapical lesions ^23^ as well as inflamed periodontal ligament around the furcation area ^24^. 

The cells in human periapical granulomas have been characterized and quantitatively analyzed in numerous studies using morphometric, immunoperoxidase and immunofluorescence techniques ^25– 32^. All these studies have shown that lymphocytes, plasma cells, macrophages and monocytes were major constituents within the periapical granuloma but only one investigated the presence of mature dendritic cells ^32^. Although the discovery of the TLRs is relatively recent, much progress has been made in our understanding of the mechanisms of innate immune recognition. TLRs recognise microbial components, trigger the innate inflammatory response and instruct the adaptive immune response. However, more research is required to answer the many remaining questions especially with regards to signalling pathways, its role in immune disorders as well as its role in infectious diseases.

The aim of the present study was to phenotypically characterize TLR2 expressing immune cells within refractory periapical granuloma using immunohistochemical and immunofluroscence expression to demonstrate the specific immune cells with toll-like receptor 2.

## Methods

The project received category-A ethical approval from the University of Otago Ethics Committee (07/210) and approval from Ngai Tahu Research Consultation Committee. 

### Sample collection and selection criteria

Periapical lesions removed during apical surgery of refractory endodontic cases between 2008 and 2010 were collected from the histopathology-archived records of the University of Otago, School of Dentistry, Oral Pathology Diagnostic Laboratory. Eight cases were selected from the sample pool of 772 for the experiment according to the selection criteria:

1. All lesions were diagnosed as periapical granuloma by registered oral pathologists.
2. All cases were treated at the postgraduate endodontic clinics, School of Dentistry, University of Otago.
3. All periapical granulomas were refractory in nature.
4. All cases had pre-operative signs and symptoms of apical periodontitis.
5. All apical surgeries and periapical biopsies were performed by registered endodontists or endodontic postgraduate students.
6. All samples had patient’s approval to be stored in the histopathology archive and used in experimental studies.
7. Previous root canal treatment met at least one of the following:
  • Previous root canal treatment appeared satisfactory on the preoperative periapical radiographs.
  • Previous root canal treatment performed by registered endodontists or endodontic postgraduate students.
  • Previous retrograde filling appeared satisfactory.
  • Satisfactory coronal seal.
  • Apical surgery was the only treatment option without jeopardizing the tooth.

Eight cases were sufficient for qualitative (descriptive) analysis and allowed a variety of tissues samples to be examined for different morphological variables. Specimens serving as positive and negative tissue controls for the immunohistochemical (IHC) study were also selected from the histopathology archive of the Medlab Dental Oral Pathology Diagnostic Laboratory. Traumatic oral ulcer tissue was used as the positive tissue control for TLR2, mucocele for CD68 (macrophage, monocyte) and lingual tonsil tissue for CD38 (lymphocyte, plasma cell) and CD83 (mature dendritic cell). Periapical scar tissue was selected as the negative tissue control.

Each histology report and the Haemotoxylin and Eosin (H & E) slides of the specimens in the archive were checked with by the oral pathologist (NF) to reconfirm their diagnosis. The details of patient’s age, sex, relevant medical and dental history, signs and symptoms of the presenting condition, pre- and post-operative periapical radiographs, clinical diagnosis and treatment received were recorded from the dental records. This information aided the case selection process. No information that could disclose a patient’s identity was recorded.

### Sample preparation

All excised lesions were fixed in 10% formalin, processed and embedded in paraffin before archival storage. 11 4 μm serial sections were cut using a microtome and individually mounted onto a positively charged slide. The first four slides were subjected to IHC staining with TLR2, CD38, CD68 and CD83 antibodies. The fifth, sixth, seventh and eighth slides were subjected to IF staining with TLR2, CD38, CD68 and CD83 antibodies and the last three slides were used for Double Immunofluorescence (DIF) staining of TLR2 expressing cells with CD38, CD68 and CD83 antibodies respectively. In addition, 4 μm section slides were made from the positive and negative tissue controls that were stained simultaneously with the experimenting slides.

### Histological examination

During routine histopathological diagnosis, each sample had at least one 4 μm section cut from three different levels of the tissue. These sections were picked up onto the same slide and subjected to Haematoxylin & Eosin (H & E) staining. The H & E stained slides were examined under light microscopy (Leica CTR5000, Leica Microsystems, Wetzlar, Germany) at x10, x20 and x40 magnification objectives. The histology report and diagnosis were made by an oral pathologist. Based on the clinical and histological findings, all samples were confirmed to be symptomatic refractory periapical granuloma. 

### Immunohistochemistry (IHC)

A pilot study was undertaken to establish optimum dilution for each antibody as well as the time required for conditioning the tissue cells in the automated slide stainer (BenchMark XT, Ventana Medical Systems, Inc. AZ, USA). Sections were picked up onto a slide, deparaffinised in xylene, dehydrated in graded alcohol, washed and then heat treated in sodium citrate buffer (pH 7.0), for 10 min at 80°C, to unmask antigens. The sections were cooled and washed in phosphate-buffered saline (PBS, pH 7.2). Endogenous peroxidase activity was quenched by incubating the sections in a solution of 3% H _2_O _2_ in methanol for 15 min. Specimens were washed with PBS and incubated in blocking agent (foetal calf serum - Sigma Aldrich – F0392 – USA) 25 μL per 4 mL of normal saline for 15 min. Sections were incubated with rabbit antihuman TLR2 polyclonal primary antibodies (abcam -ab24192- Cambridge, UK) at 1:500 to 1:700 dilution for 30 min at 25°C. The sections were then washed in PBS and incubated with secondary goat anti-rabbit and goat anti-mouse immunoglobulins in phosphate buffered saline (PBS) using the LSAB2-HRP link system (DakoCytomation – K0675 - Carpinteria, CA, USA).

The manufacturer’s instructions were followed for the sequential incubation and durations for the exposure to the secondary antibodies. After washing with PBS, the sections were incubated with diaminobenzidine substrate kit (K3468 - Dako, Carpinteria, CA, USA) that resulted in a brown-coloured precipitate at the antigen–antibody binding sites. Finally, all the sections were washed in deionized water, dehydrated and mounted on individual slides. Positive (traumatic oral ulcer) and negative controls (periapical scar) as well as experimental sections with no primary antibody were included in all immunohistochemical runs.

The preparation of the sections for immunohistochemistry staining with antibodies CD38, CD68 and CD83 was similar to the TLR2 antibody technique. The dilutions used for monoclonal mouse antihuman CD38 (abcam -ab49644- Cambridge, UK), CD68 (abcam -ab955- Cambridge, UK), and CD83 (abcam -ab49324- Cambridge, UK) primary antibodies (Dako) were 1:50-1:100, 1:200-1:400, and 1: 20-1:40 respectively. The duration of incubation was 2 hours for both the primary antibodies. The secondary antibody system used by the automatic immunostainer was polymer-based Universal HRP multimer, which was prediluted and ready to be used in dispensers (#760-500 ultra- View™; Ventana Medical systems Inc.). Finally, the sections were incubated with universal alkaline phosphatase red detection chromogen kit (#760-501 ultraView™; Ventana Medical systems Inc.), washed in deionized water, dehydrated and mounted. Positive (lingual tonsils or mucocele) and negative controls (periapical scar) as well as experimental sections with no primary antibody were included in all immunohistochemical runs. Primary antibodies are listed in Table 1 & Table 2.

**Table 1.** Primary antibodies used in immunohistochemistry pilot study.

Primary Antibody	Manufacturer	Raised in	React with	Colonality	Concentration (mg/ml)	Recommended dilution range	Target
TLR2	abcam (ab24192) Cambridge, UK	Rabbit	Human	Polyclonal	1	1:500 – 1:700	#
CD38	abcam (ab49644) Cambridge, UK	Mouse	Human	Monoclonal	*	1:50 – 1:100	Lymphocyte Plasma cell
CD68	abcam (ab955) Cambridge, UK	Mouse	Human	Monoclonal	0.04	1:200 – 1:400	Macrophage Monocyte
CD83	abcam (ab49324) Cambridge, UK	Mouse	Human	Monoclonal	1	1:20 – 1:40	Mature dendritic cell

**Table 2.** Primary antibodies used in immunohistochemistry.

Primary Antibody	Target	Dilution	Control			Cell Conditioning Time
			Positive Tissue	Negative Tissue	Negative Antibody	
TLR2	Toll-like Receptor	1:700	Traumatic Oral Ulcer	Periapical Scar	Rabbit Isotype IgG 1:500	Standard (60 minutes)
CD38	Lymphocytes Plasma Cell	1:100	Lingual Tonsil	Periapical Scar	Mouse Isotype IgG 1:500	Standard (60 minutes)
CD68	Macrophage Monocyte	1:200	Mucocele	Periapical Scar	Mouse Isotype IgG 1:500	Standard (60 minutes)
CD83	Mature Dendritic Cell	1:20	Lingual Tonsil	Periapical Scar	Mouse Isotype IgG 1:500	Standard (60 minutes)

### Immunofluroscence (IF)

A pilot study was undertaken to establish optimum dilution for each antibody as well as the time required for conditioning the tissue cells in an automated slide stainer (BenchMark XT, Ventana Medical Systems, Inc. AZ, USA) which was used only for the deparafinisation and cell conditioning standard steps. The primary antibodies were Alexa Fluor ^®^ 488 (Invitrogen -A-11008- Carisbad, CA, USA) goat antirabbit for TLR2 green immunofluroscence staining and Alexa Fluor ^®^ 594 (Invitrogen -A-21203- Carisbad, CA, USA) donkey antimouse red immunofluroscence staining for CD38, CD68 and CD83. Positive controls (traumatic oral ulcer for TLR2; lingual tonsils and mucocele for CD38, CD68 and CD83), negative controls (periapical scar) as well as as experimental sections with no primary antibody were included in IF protocol.

All slides were manually mounted with Vectashield ^®^ -aqueous hard set mountant (H- 1000 - Maravai LifeSciences) with nucleus counterstain DAPI (4’,6-diamidino-2-phenylindole, stains nucleus in florescent blue) in a fume cupboard. Slides were examined immediately and photographs taken within a 48 hour period under the immunofluorescence microscope (Lecia M205 FCA). Slides were kept in a 4° C light-tight fridge if not viewed immediately. The optimum primary and secondary antibody incubations were investigated, determined in pilot studies and summarized in Table 2 & Table 3. Primary antibody incubation was at 4°C overnight (18–24 hours) and secondary antibody incubation (Alexa Fluor ^®^) at room temperature for one hour in complete darkness.

**Table 3.** Immunofluorescence protocols for primary antibodies.

Secondary Labeling Antibody	Manufacturer	Raised in	React with	Recommended dilution range	Fluorescence	Targeted primary antibody (cells)
Alexa Fluor® 488	Invitrogen (A-11008) Carisbad, CA, USA	Goat	Rabbit	1:200 – 1:2000	Green	TLR2
Alexa Fluor® 594	Invitrogen (A-21203) Carisbad, CA, USA	Donkey	Mouse	1:200 – 1:2000	Red	CD38 (lymphocyte, plasma cell) CD68 (macrophage) Cd83 (matured dendritic cell)

To ensure high quality images that reflect the observation, two of the three controls in the camera setting were set with a fixed value: Gain 3.0 (range between 0.6 – 6.0) and Offset -500 (range between -1100 – +2995). The exposure time measured in milliseconds (ms) was the only adjustable camera setting. This was determined for each fluorochrome at each magnification power ( Table 4).

**Table 4.** Exposure time for immunofluorescence imaging.

Secondary Antibody	Magnification		
	x200	x400	x1000
Alexa Fluor 488®	700 – 900 ms	600 – 800ms	300 – 500 ms
Alexa Fluor 594®	250 – 400 ms	200 – 350ms	150 – 250ms

### Double immunofluroscence (DIF)

The parameters of the DIF staining procedure were determined by the IF pilot study. However, the primary antibodies were pre-mixed into a cocktail solution (without changing their optimum dilutions) and incubated simultaneously. This pre-mixing and simultaneous incubation also applied to the secondary labeling antibodies.

### Qualitative analysis

***Histology and immunohistochemistry (IHC).*** All H & E and IHC stained sections were viewed under a light microscope (Leica DM5000B, Leica Microsystems, Wetzlar, Germany) under magnifications up to x100 objective. A cell was determined as immuno-positive when it demonstrated distinctive brown stain on the cell membrane and/or cytoplasm around a nucleus. Images were taken using a CCD camera (Leica DC500, Leica Microsystems, Wetzlar, Germany), mounted on the microscope, controlled by computer software ( Leica FireCam Version 1.5, Leica Microsystem, Heerbrugg, Switzerland).

***Immunofluroscence (IF) and double immunofluorescence (DIF).*** All IF stained sections were viewed under a fluorescence microscope (Olympus AX70, Olympus Corporation, Center Valley, PA, USA) under magnifications up to x100 objectives. Images were taken using the CMOS camera (Go-3, QImaging, Surrey, BC, Canada) mounted on the microscope and controlled by computer software ( Macintosh QCapture Suite, 2.98.2 QImaging, Surrey, BC, Canada). A cell was counted as positive when it demonstrated distinctive fluorescence on the cell membrane and/or cytoplasm surrounding the nucleus. Since the fluorescence microscope only observes one wavelength at a time, the separately labeled protein target and the nucleus cannot be observed simultaneously. To overcome this problem Photoshop (CS5 – 12.0 – White Rabbit - Adobe Systems Incorporated, San Jose, CA, USA) software was employed for qualitative analysis. An area of interest was photographed under different wavelength with the slide remaining stationary. Images were superimposed and screened using the Photoshop software to disclose positive cells.

Qualitative analysis of the DIF followed the same principles as IF. A cell was identified to co-express two targeted proteins when the superimposed and screened images showed both green and red fluorescence on the cell membrane and/or cytoplasm. The objective of the DIF qualitative analysis was to identify TLR2 expressing cells as lymphocytes/plasma cells (CD38), Macrophages/monocytes (CD68) and/or mature dendritic cells (CD83).

## Results

### Histological examination

**Figure 1.**
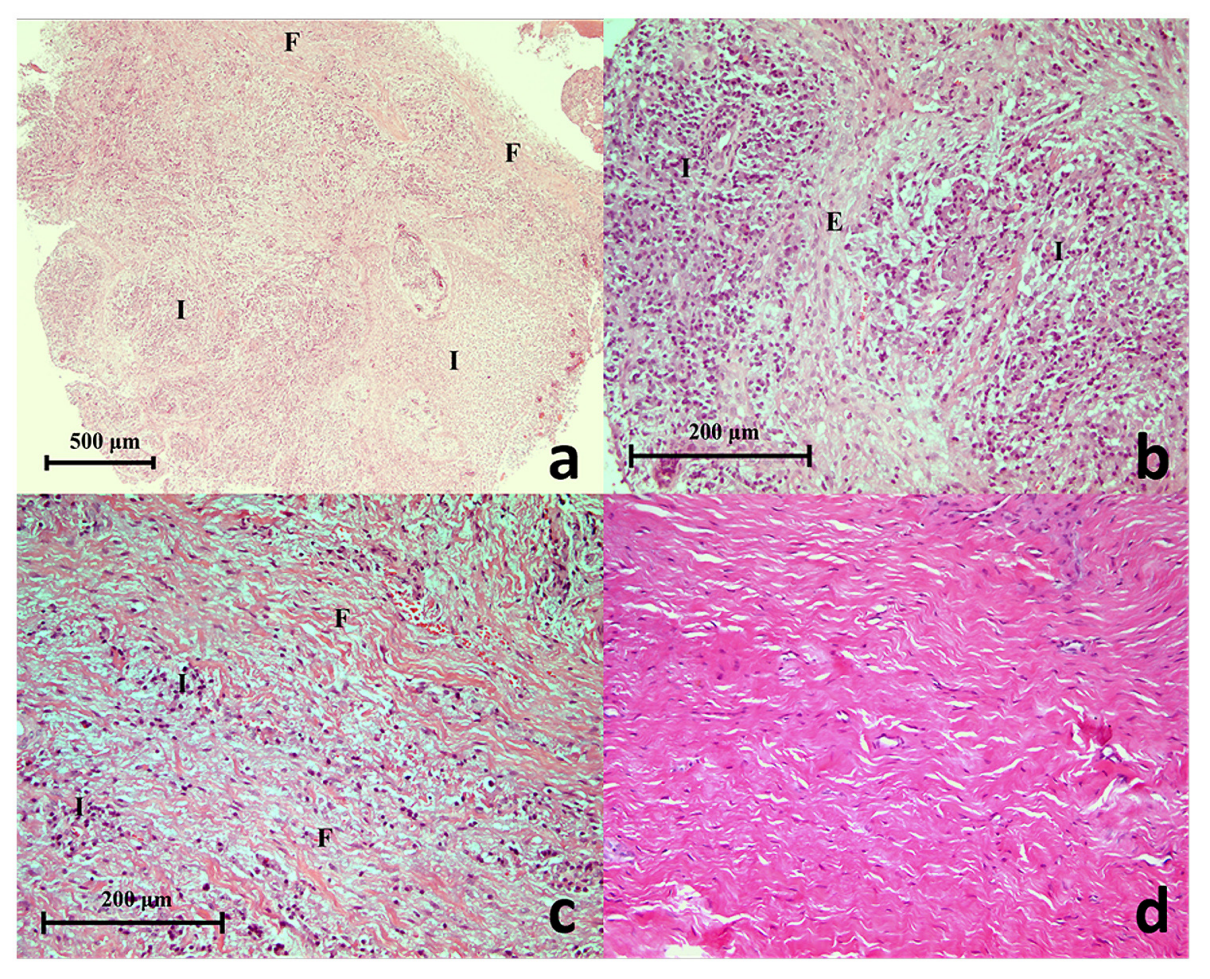
( **a**) A histopathology section of a selected refractory periapical granuloma showing areas of fibrous connective tissue (F), blood vessels, inflammatory cells (I) and interspersed odontogenic epithelium (Haematoxylin & Eosin staining x50), ( **b**) Proliferating epithelial cells (E) surrounded by chronic inflammatory cells (I) (Haematoxylin & Eosin staining x200), ( **c**) Fibrous connective tissue (F) with moderate chronic inflammatory cell infiltrate (I) (Haematoxylin & Eosin staining x200), ( **d**) Histopathology section of a periapical scar showing the un-inflamed, relatively acellular and avascular dense collagen tissue (Haematoxylin & Eosin staining x200).

The routine diagnostic H & E stained sections of the selected periapical granuloma lesions were retrieved from the histopathology-archived records. All tissue sections showed characteristics of granulation tissue ( Figure 1a, b, c), typically mature fibrous connective tissue with a moderately intense infiltrate of chronic inflammatory cells dominated by lymphocytes. Occasionally, strands of stratified squamous epithelium of odontogenic origin (epithelial rests of Malassez) were found interspersed in the granulation tissue of some lesions. In the periapical scar (negative tissue control) inflammatory cells were absent and the lesion was characteristically acellular, with the exception of fibroblasts associated with collagen, with a dense avascular collagen structure ( Figure 1d).

### Immunohistochemistry

**Figure 2.**
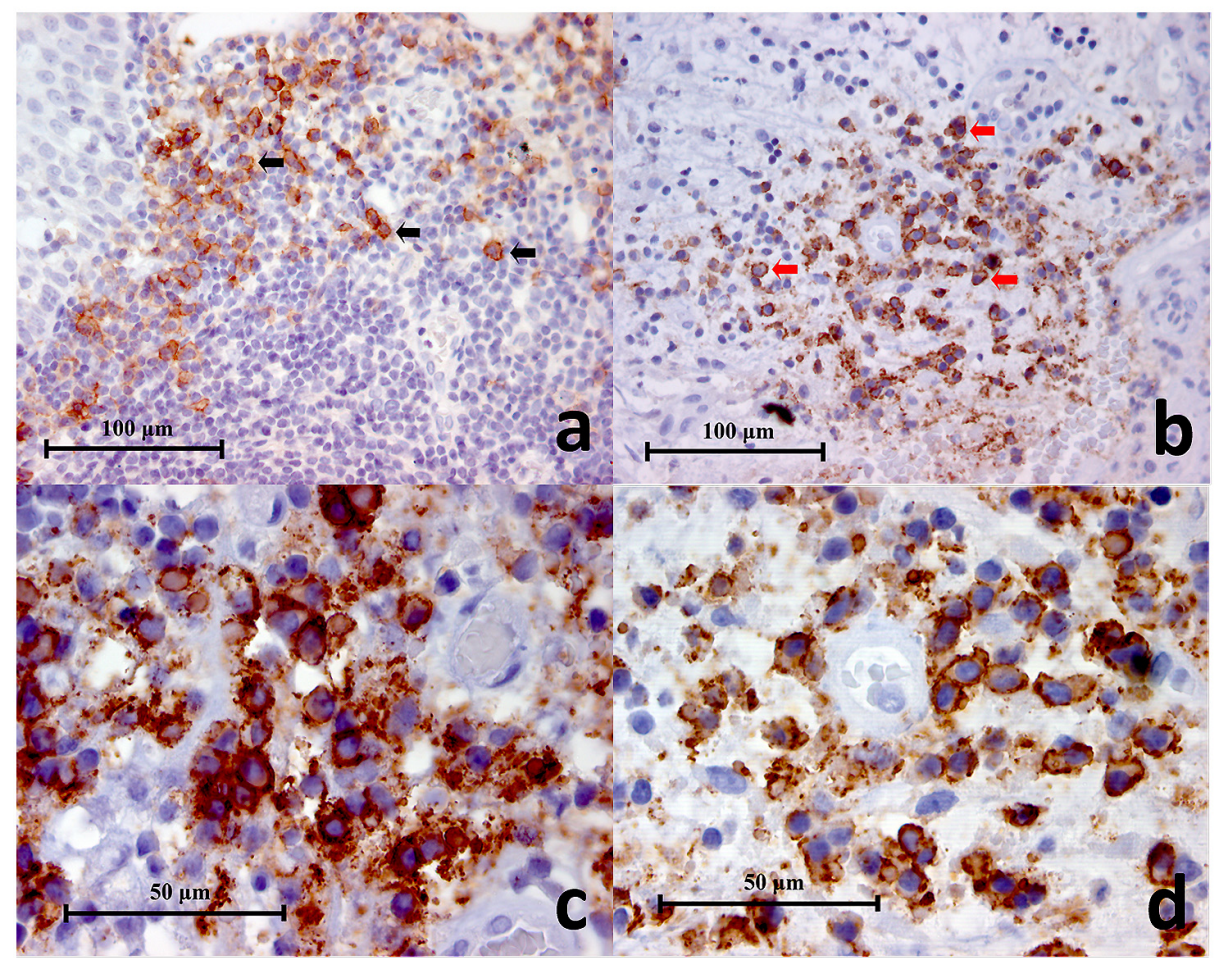
( **a**) Representative histopathology section of positive control (lingual tonsil) showing CD38+ cells (black arrows) in the germinal centres (CD38 Immunohistochemistry x400), ( **b**) Representative histopathology section from a periapical granuloma showing a cluster of CD38+ cells (red arrows) (CD38 Immunohistochemistry x400), ( **c, d**) Two images of CD38+ cells under high power magnification showing characteristic lymphocyte and plasma cell circular or oval shape with staining mainly on the cell membrane (CD38 Immunohistochemistry x1000).

***CD38.*** In the lingual tonsil section (positive control), clusters of lymphocytes within the germinal centres were positively stained and appeared as small circular or oval brown cells that were closely packed together ( Figure 2a). All the periapical granuloma samples showed CD38 ^**+**^ cells and had the same staining pattern as the CD38 ^**+**^ cells in the lingual tonsil. These CD38 ^**+**^ cells dominated the inflammatory cell infiltrate and were mostly found in large clusters evenly distributed in the granulation tissue with some individual positive cells scattered in between ( Figure 2a, b). A closer look of the CD38+ cells under high power magnification (x1000) revealed that the brown stains were mainly located on the cell membrane ( Figure 2c, d). However since the surrounding cytoplasm could be quite narrow in width it was not easy to distinguish under low power magnifications (x200 and x400). The CD38 ^**+**^ cells are roughly 7 to 10 μm in diameter. In the negative controls no positive staining was found.

**Figure 3.**
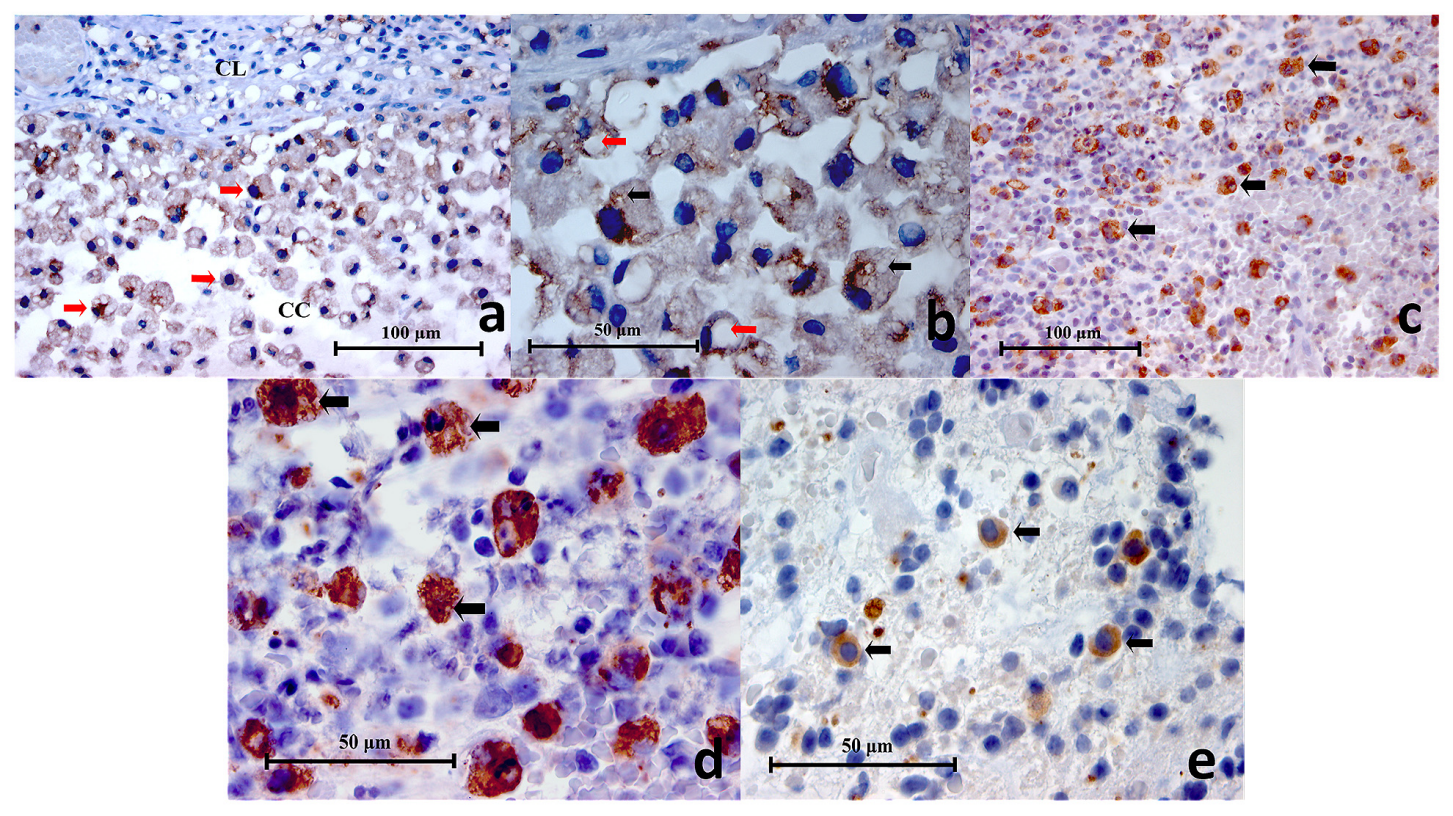
( **a**) Representative histopathology section of a mucocele showing positively stained macrophages (red arrows), cystic lining (CL) and cystic cavity (CC) (CD68 Immunohistochemistry x400), ( **b**) High power magnification of CD68+ cells in a mucocele histopathology section. Cytoplasmic vesicles (black arrows) and phagosomes (red arrows) are clearly demonstrated (CD68 Immunohistochemistry x1000), ( **c**) Representative histopathology section of a symptomatic periapical granuloma showing CD68+ cells which stained brown (black arrows) (CD68 Immunohistochemistry x400), ( **d**) High power magnification of the larger CD68+ cells showing spongy appearances and wavy cellular outlines which resembled macrophages. Positive immunostaining is located on the cell membrane as well as the cytoplasm. Unstained intracellular vesicles can be identified (black arrows) (CD68 Immunohistochemistry x1000), ( **e**) High power magnification of the smaller CD68+ cells showing a rounded cellular profile which resembled monocytes (black arrows) (CD68 Immunohistochemistry x1000).

***CD68.*** In the mucocele (positive control), CD68 ^**+**^ cells appeared as large globular spongy/foamy brown cells, roughly 20 μm in dimension ( Figure 3a). They were found adjacent to the cyst lining inside the cyst cavity. Under high power magnification (x1000) small cytoplasmic vesicles and large phagosomes were observed which are also characteristic of macrophages ( Figure 3b). The brown stain was mainly located on the cell membrane and cytoplasm. 

In the periapical granuloma lesions, five out of eight samples were identified to contain CD68 ^**+**^ cells ( Figure 3c). CD68 ^**+**^ cells identified in the five periapical granuloma lesions were abundant but were mainly located in localized areas of the granulation tissue. The remaining three samples did not show distinctive positive staining. Furthermore, two morphologically distinctive cells were observed in the periapical granulomas; large spongy cells that measured up to 20 μm across with irregular wavy outline that appeared to be macrophages ( Figure 3d) and small circular cells that were about 10 μm in diameter which resembled monocytes ( Figure 3e). Under high power magnification (x1000) small cytoplasmic vesicles were observed within the macrophages. These vesicles were not stained and stood out from the stained cell membrane and cytoplasm and thus contributed to the spongy appearance. No positive staining was found in the negative controls.

**Figure 4.**
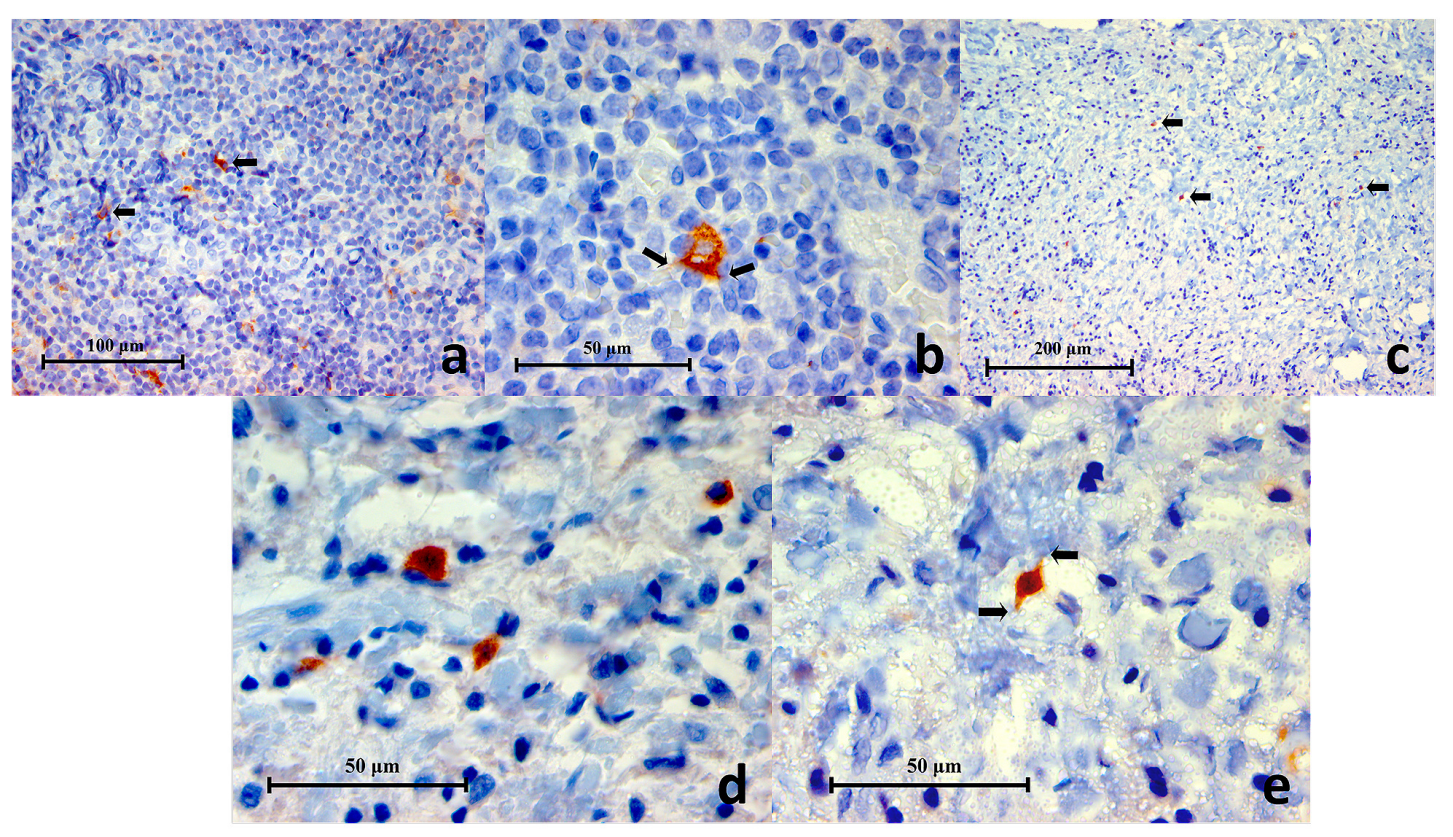
( **a**) Representative histopathology section of a germinal centre within lingual tonsil demonstrating CD83+ cells (black arrows) (CD83 Immunohistochemistry x400), ( **b**) High power magnification of a CD83+ cell showing a polygonal outline with little thorn-like or filamentous extensions (black arrows) characteristic of a dendritic cell (CD83 Immunohistochemistry x1000), ( **c**) Representative histopathology section from a periapical granuloma showing CD83+ cells (black arrows). Characteristically these cells were found in low numbers within a lesion (CD83 Immunohistochemistry x200), ( **d, e**) Two images of high power magnification of CD83+ cells in a periapical granuloma showing some examples of the variable cellular morphology they exhibited. Little thorn-like extensions can be seen on some cells (black arrows) indicative of dendritic cells (CD83 Immunohistochemistry x100).

***CD83.*** In the lingual tonsil (positive control) sample, CD83 ^**+**^ cells were sparingly scattered throughout the germinal centres ( Figure 4a). These cells exhibited variable morphology, being circular, oval or asymmetrical polygonal cells. In addition, some CD83 ^**+**^ cells showed small thorn-like or filamentous extensions under high power magnification ( Figure 4b), which is characteristic of dendritic cells. The positive immunohistochemical stain was observed on the cell membrane and the cytoplasm. In the periapical granuloma samples, CD83 ^**+**^ cells were not found in two samples and appeared to be rare in another two samples. In the remaining four samples CD83 ^**+**^ cells were readily detected, however, they constituted a very small population in the inflammatory cell infiltrate ( Figure 4c). The size of the CD83 ^**+**^ cells were variable depending on the out stretch of cytoplasmic extensions. The cell body may be 10 μm in diameter and the cytoplasmic extensions up to 5 μm have been observed ( Figure 4d, e). All negative controls showed negative immunostaining.

**Figure 5.**
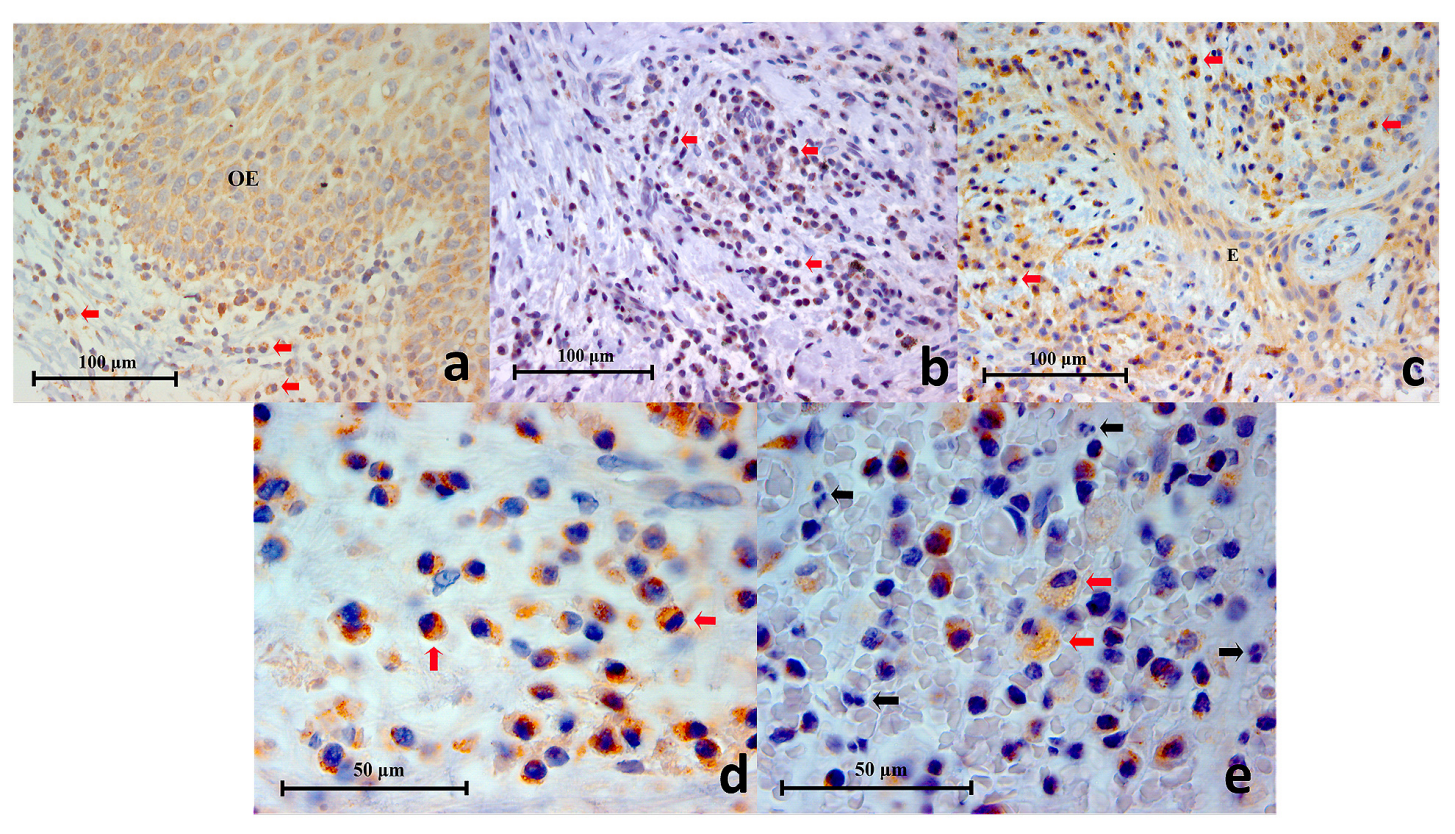
( **a**) Representative histopathology section of a traumatic oral ulcer showing an area of intact oral epithelium (OE) which was positively stained for TLR2. Mononuclear inflammatory cells (red arrows) in the connective tissue are also stained brown (TLR2 Immunohistochemistry x400), ( **b**): Representative histopathology section from a periapical granuloma showing numerous mononuclear inflammatory cells stained brown (red arrows), demonstrating TLR2 expression (TLR2 Immunohistochemistry x400), ( **c**) Representative histopathology section from a periapical granuloma showing TLR2+ immunostaining on numerous mononuclear inflammatory cells (red arrows) as well as interspersed odontogenic epithelium (E) (TLR2 Immunohistochemistry x400), ( **d**) Image of TLR2+ cells resembling immune cells of lymphoid lineage under high power magnification (red arrows). Note the cell membrane and cytoplasm are stained brown (TLR2 Immunohistochemistry x1000), ( **e**) High power magnification of a periapical granuloma histopathology section showing large foamy TLR2+ cells resembling macrophages (red arrows). Note that the neutrophils (black arrows), characterised by multi-lobed nuclei, were negative to TLR2 immunostaining (TLR2 Immunohistochemistry x1000).

***TLR2.*** In the positive control, traumatic oral ulcer tissue, various mononuclear inflammatory cells and the intact oral epithelial cells were stained brown on their cell membrane and cytoplasm, demonstrating TLR2 expression ( Figure 5a). Similarly, all periapical granuloma samples showed various mononuclear inflammatory cells displaying the same staining pattern observed in the traumatic oral ulcer (positive control) ( Figure 5b, c). The most abundant TLR2 ^**+**^ cells in the periapical granuloma where small circular or oval cells of about 7 to 10 μm in diameter and appeared to be lymphocytes ( Figure 5d). In some samples, TLR2 ^**+**^ cells exhibited foamy appearances which resembled macrophages ( Figure 5e). These foamy cells are roughly 12 to 15 μm across, almost double in size compared to lymphocytes. Occasionally, interspersed odontogenic epithelial cells also stained brown, suggesting TLR2 positivity. On the other hand, neutrophils, red blood cells (RBC) and collagen ground substance were not stained. In the negative controls no cells or structures showed TLR2 immunostaining.

### TLR2 IF on experiment controls

In the positive control tissue, various inflammatory cells were observed to fluoresce on the cell surface and in the cytoplasm. The oral epithelium also expressed TLR2 and was identified by its location, distinct morphology and cellular arrangement. Red blood cells (RBC) were identified by their autofluorescent glow, biconcave cellular profile and the absence of a nucleus. All negative controls did not show positive fluorescence.

### CD38 IF on experiment controls

**Figure 6.**
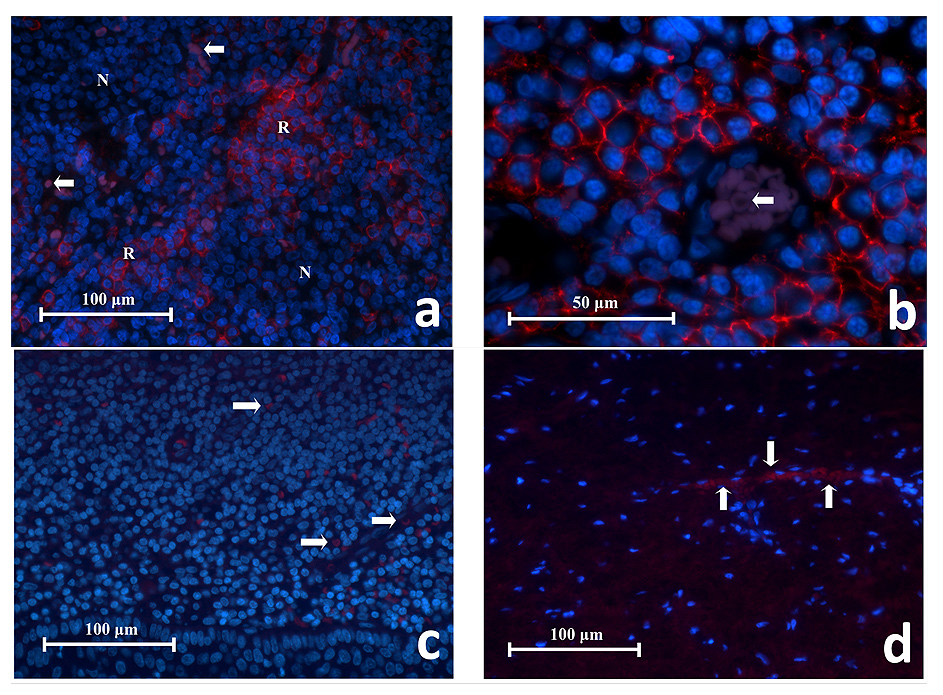
( **a**) Representative histopathology section of CD38 ^+^ cells in lingual tonsil tissue showing collections of red fluorescing cells (R) separated by areas of CD38 ^-^ cells that had no red fluorescence (N). RBCs had faint purple autoflorescence (white arrows) (CD38 Immunofluroscence x400). ( **b**) High power magnification of CD38 ^+^ cells in lingual tonsil tissue. These cells have a circular or oval profile with a narrow space of cytoplasm surrounding the nucleus, consistent with being lymphocytes. The red fluorescence is located on the cell membrane whereas the cytoplasm was negative. These cells are closely associated to one another. This image also shows a blood vessel containing RBCs which glowed in faint purple (white arrow) (CD38 Immunofluroscence x1000). ( **c**) Representative section of negative antibody control. ( **d**) Representative section of negative tissue control (periapical scar) (x400). Note the red fluorescing cells represent RBCs (white arrows) which exhibit autofluorescence and were scattered in the tissue or within the blood vessels.

Some lymphocytes within the germinal centres of the lingual tonsil showed CD38 positivity. These CD38 ^**+**^ cells were small and had a circular or oval profile ( Figure 6a). They appeared to be tightly packed to each other, arranged in clusters separated by the CD38 ^**-**^ cells and appeared as collections of red fluorescent rings. Under high power magnification, the red fluorescence was mainly located on the cell membrane ( Figure 6b). The cytoplasm typically was not stained and appeared as a thin black space surrounding the nucleus. The size of the CD38 ^**+**^ cells was about 10 to 13 μm in diameter. Red blood cells (RBCs) were identified by their smaller size (about 7 μm across), faint purple autofluorescent glow, biconcave cellular profile and the absence of a nucleus. All negative controls showed negative fluorescence ( Figure 6c, d).

### CD68 IF on experiment controls

**Figure 7.**
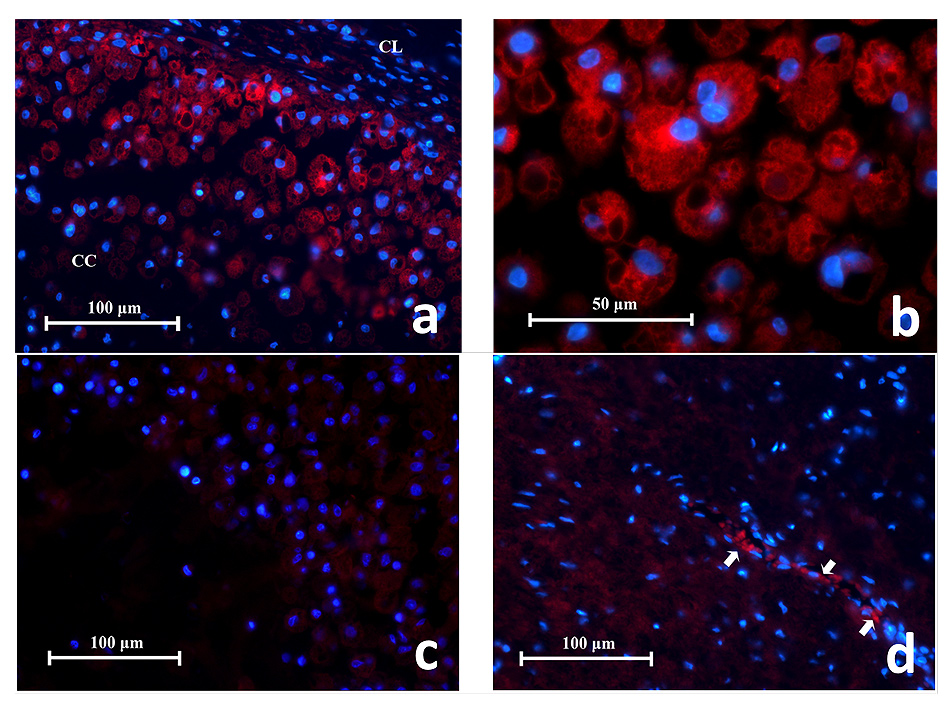
( **a**) Representative histopathology section showing CD68 ^+^ cells in a mucocele fluorescing in red. They are found in large number adjacent to the cystic lining (CC) and spread into the cystic cavity (CC). Characteristics of macrophage cells are evident e.g. irregular cell shape, intra-cellular vacuoles (x400). ( **b**) High power magnification of CD68 ^+^ cells in a mucocele showing their large spongy globular appearance. The red fluorescence is observed on the cell membrane and cytoplasm but not the intracellular vesicles or phagosomes (x1000). ( **c**) Representative section of negative antibody control. ( **d**) Representative section of negative tissue control (periapical scar) with autofluorescing RBCs (white arrows) (x400).

Macrophages in the mucocele expressed CD68 and appeared as large red fluorescent spongy cells measured up to 25 μm across ( Figure 7a, b). They were found in large numbers in the cystic cavity just beneath the epithelial lining. Under high power magnification the red fluorescence was located on the cell membrane as well as the cytoplasm. Also observed were the intracellular vesicles and phagosomes which contributed to the spongy appearance of CD68 ^**+**^ cells ( Figure 7b). The negative controls showed no fluorescence ( Figure 7c, d).

### CD83 IF on experiment controls

**Figure 8.**
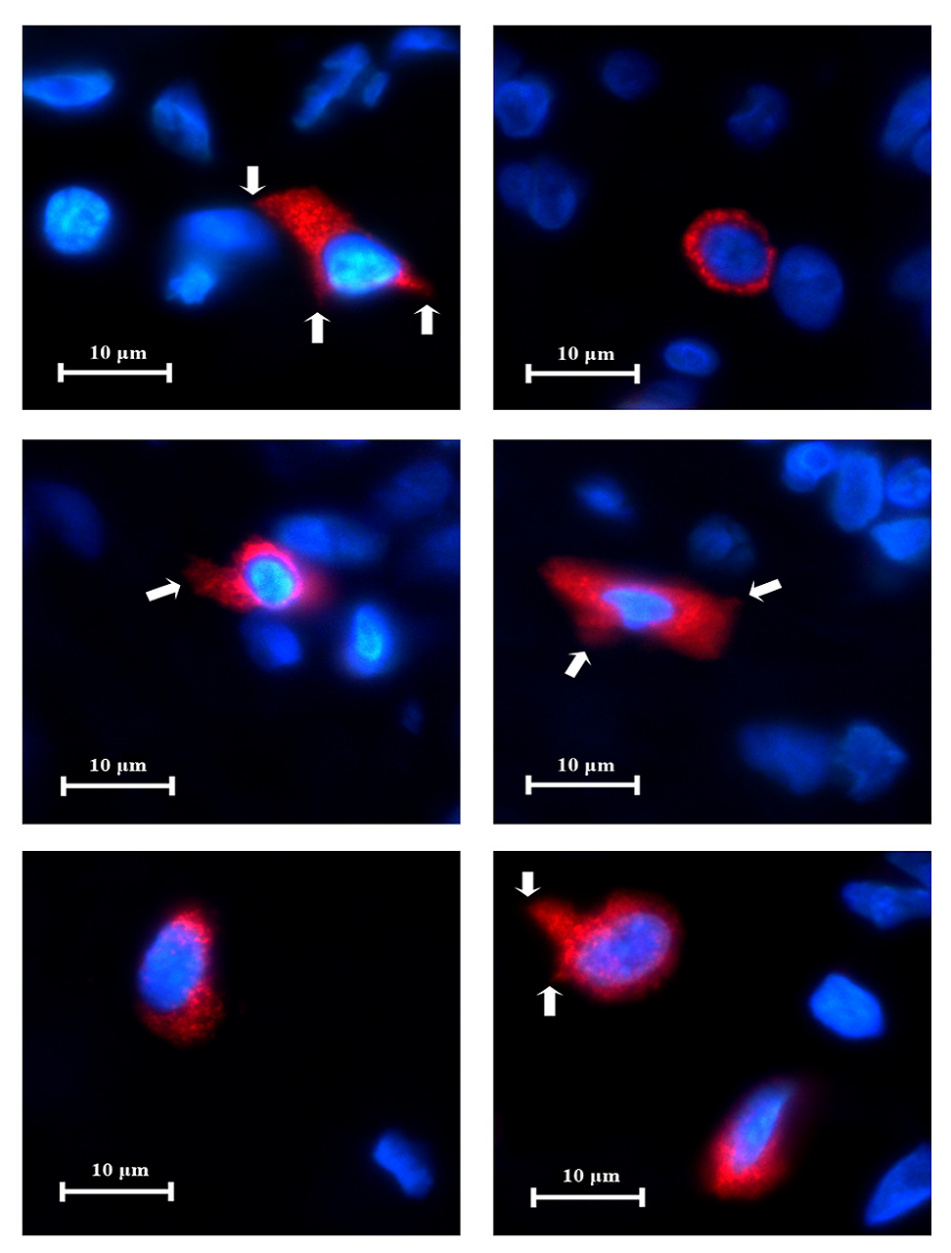
High power magnification of CD83+ cells found in the lingual tonsil tissue. The CD83 ^+^ cells exhibit several cellular shapes: circular, oval, teardrop and asymmetric polygons. Occasionally, little thorn-like projections could be detected on cell surfaces (white arrows). These characteristics are typical of dendritic cells (x1000).

In the lingual tonsil tissue, CD83 ^**+**^ cells were a minority group amongst resident cells. Some CD83 ^**+**^ cells were located within germinal centres, connective tissue or in between minor salivary glands. These cells displayed red fluorescence on the cell membrane and cytoplasm. They exhibited several cellular outlines including circular, oval, teardrop or asymmetric polygons ( Figure 8). Under high power magnification, little thorn-like projections were sometimes seen extending from the cell surface which is a characteristic of dendritic cells. The cell body of the CD83+ cells are roughly 10 μm in diameter with the cytoplasmic extensions measuring up to 5 μm in length ( Figure 8). All negative controls did not show fluorescence.

### Double immunofluorescence (DIF) experiment

The cellular characteristics and distribution of each cell marker in the DIF experiments were similar to IF. In all samples of periapical granulomas, the majority of inflammatory cells and odontogenic epithelium expressed TLR2. CD38 ^**+**^ cells (lymphocytes) remained the most abundant inflammatory cells in all the periapical granuloma lesions followed by CD68 ^**+**^ cells (macrophage). In contrast, CD83 ^**+**^ cells (dendritic cells) were few or rare in the samples. 

### CD38 and TLR2 DIF

**Figure 9.**
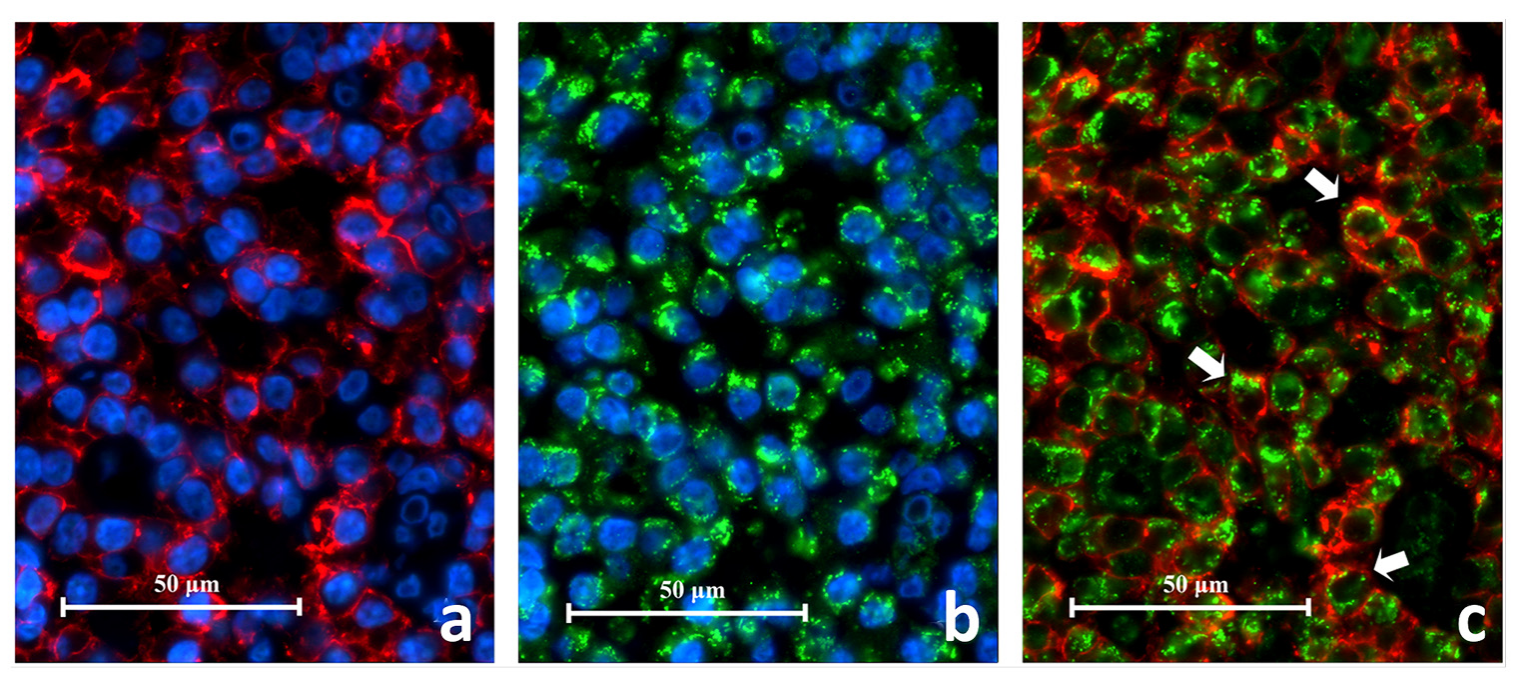
High power magnification images of a periapical granuloma captured under different emitting fluorescence. Image **A** shows CD38+ cells with red fluorescing cell membrane and unstained cytoplasm. Image **B** shows TLR2+ cells displayed green fluorescence on the cell membrane as well as on the cytoplasm. The superimposed image **C** reveals CD38+/TLR2+ cells which have orange-red cell membrane and green cytoplasm (white arrows) (CD38/TLR2 Double Immunofluorescence x1000).

CD38 ^**+**^ cells expressing TLR2 ^**+**^ (CD38 ^**+**^/TLR2 ^**+**^) were identified in all eight periapical granuloma samples and were abundant throughout the lesion. Under high power magnification CD38 was located on the cell membrane with the cytoplasm left unstained ( Figure 9). On the other hand, the TLR2 green fluorescence appeared to have stained both the cell membrane and cytoplasm. The CD38 ^**+**^/TLR2 ^**+**^ cells had a circular profile that resembled lymphocytes in the lingual tonsil and were closely packed together like soap bubbles. These cells displayed a thin orange-red cell membrane which enclosed the narrow green cytoplasm which surrounded the nucleus. The results indicate that lymphocytes express TLR2 in refractory periapical granuloma lesions.

### CD68 and TLR2 DIF

**Figure 10.**
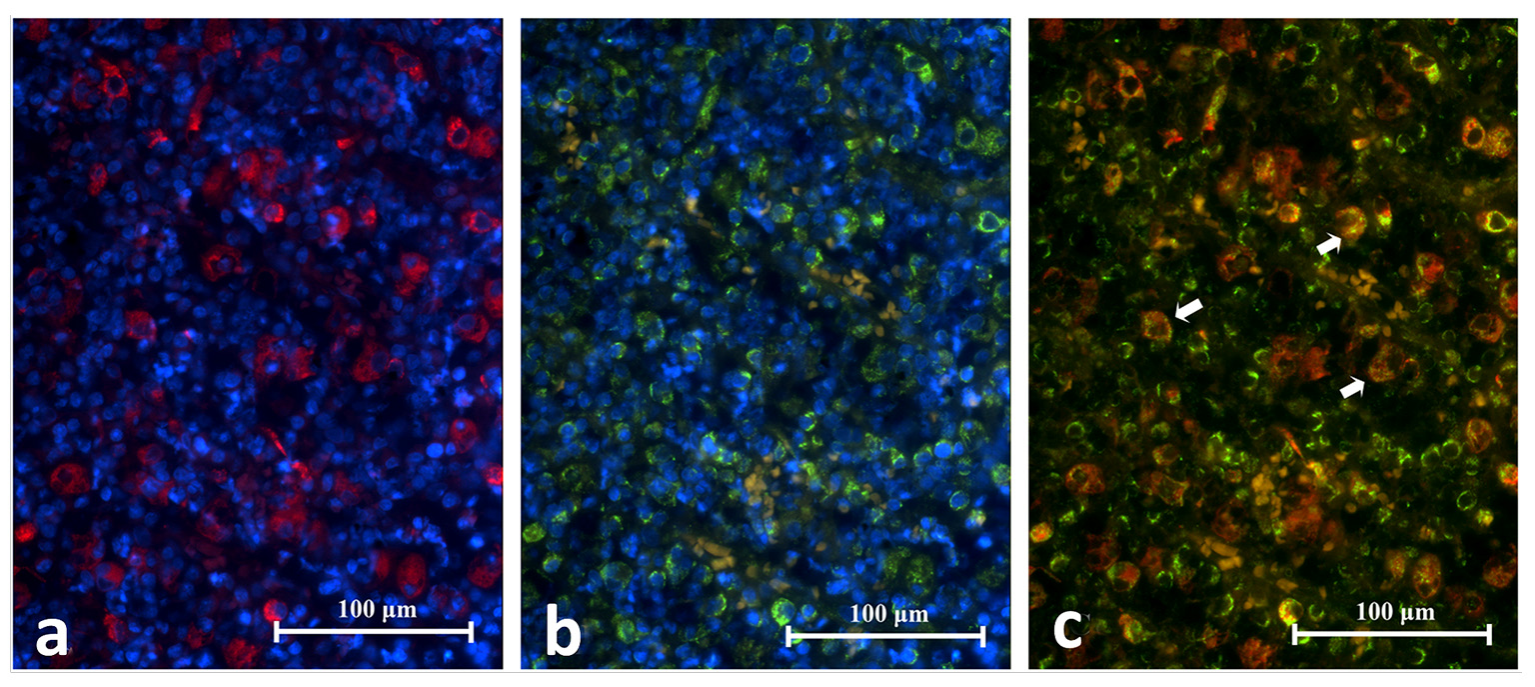
Representative histopathology section of the same area in a periapical granuloma showing images captured under different emitting fluorescence’s. Image **A** shows CD68+ cells fluorescing in red and **B** shows TLR2+ cells fluorescing in green. The superimposed image **C** reveals the CD68+/TLR2+ cells (white arrows) (CD68/TLR2 Double Immunofluorescence x400).

**Figure 11.**
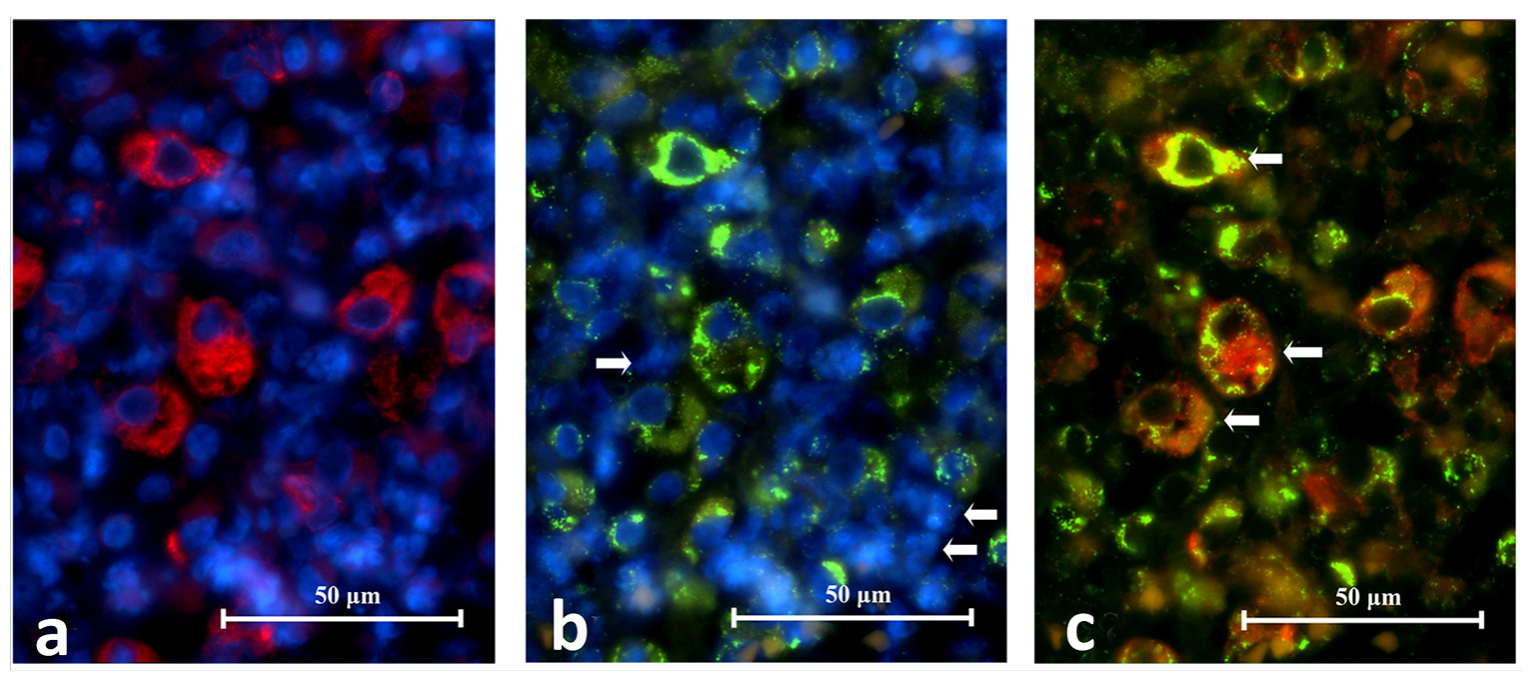
High power magnification images of a periapical granuloma captured under individual emitting fluorescence. Image **A** shows CD68+ cells fluorescing in red. Notice the cell membrane and cytoplasm are stained but not intracellular vesicles. Image **B** shows TLR2+ cells fluorescing in green. Notice the multi-lobular nuclear cells resembling neutrophils were negative to TLR2 staining (white arrows). The superimposed image **C** reveals CD68+/TLR2+ cells which have cell membrane and cytoplasm fluorescing in orange or a mixture of red and green speckles (white arrows) (CD68/TLR2 Double Immunofluorescence x1000).

The co-expression of CD68 and TLR2 on cells was identified in five periapical granuloma samples. Under high power magnification both CD68 and TLR2 fluorescence stained the cell membrane and cytoplasm but not the intracellular vesicles. The CD68 ^+^/TLR2 ^+^ cells fluoresced as a mixture of orange, red and green. The CD68 ^+^/TLR2 ^+^ cells were large and had spongy globular profiles that resembled macrophages in the mucocele. They were abundant but were found in localized areas of a lesion *i.e.* they were not dispersed throughout a lesion ( Figure 10). Many RBCs were found interspersed in the area. These RBCs autofluoresced in purple and yellow on the red and green images respectively. Although they may appear as orange cells in an overlapped image, they are distinguished by their smaller size, biconcave profile and lack of nucleus ( Figure 11). 

### CD83 and TLR2

**Figure 12.**
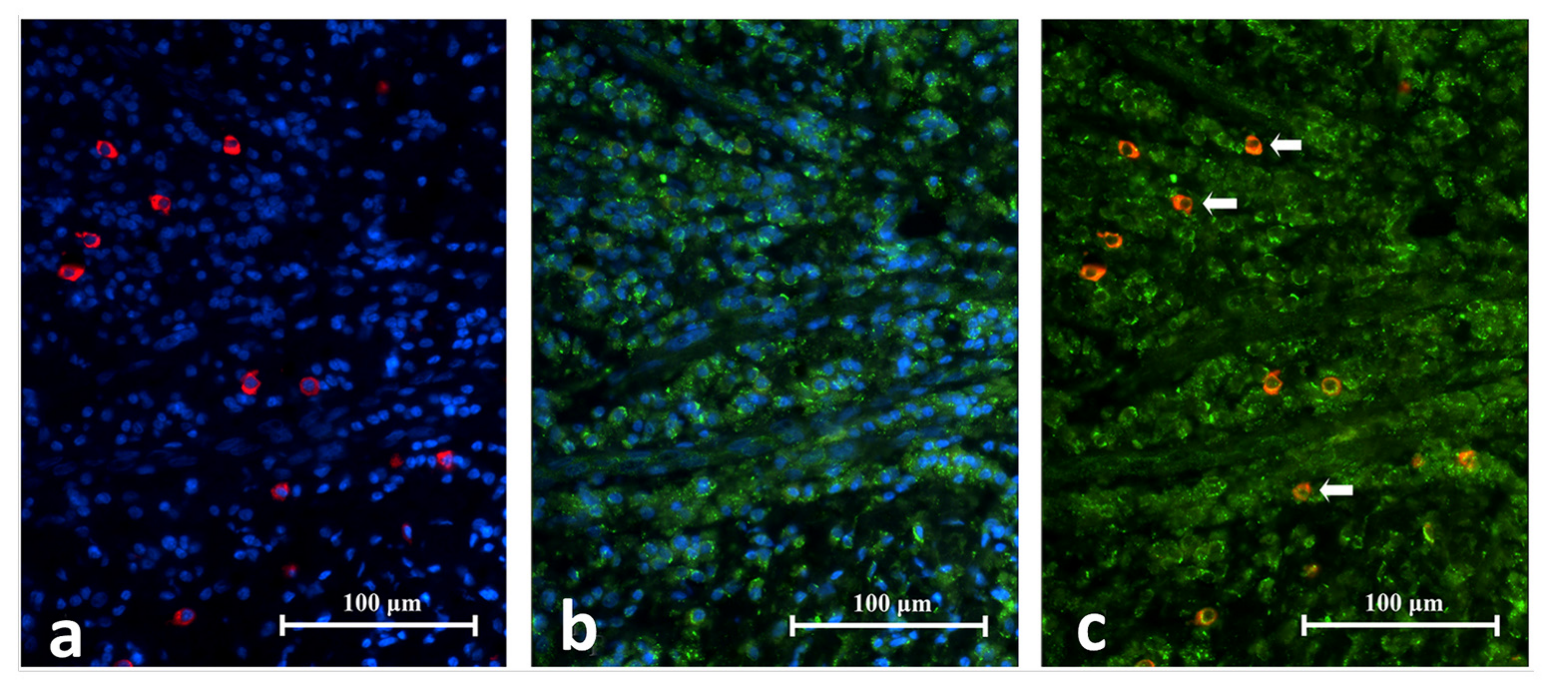
Representative histopathology section of the same area in a periapical granuloma showing images captured under individual fluorescence. Image **A** shows CD83+ cells fluorescing in red. Notice the cells have variable outlines and cytoplasmic extension. They contributed as a minor cell population in the periapical granuloma. Image **B** shows TLR2+ cells fluorescing in green. The superimposed image **C** reveals the CD83+/TLR2+ cells in orange-red fluorescence (white arrows) (CD83/TLR2 Double Immunofluorescence x400).

**Figure 13.**
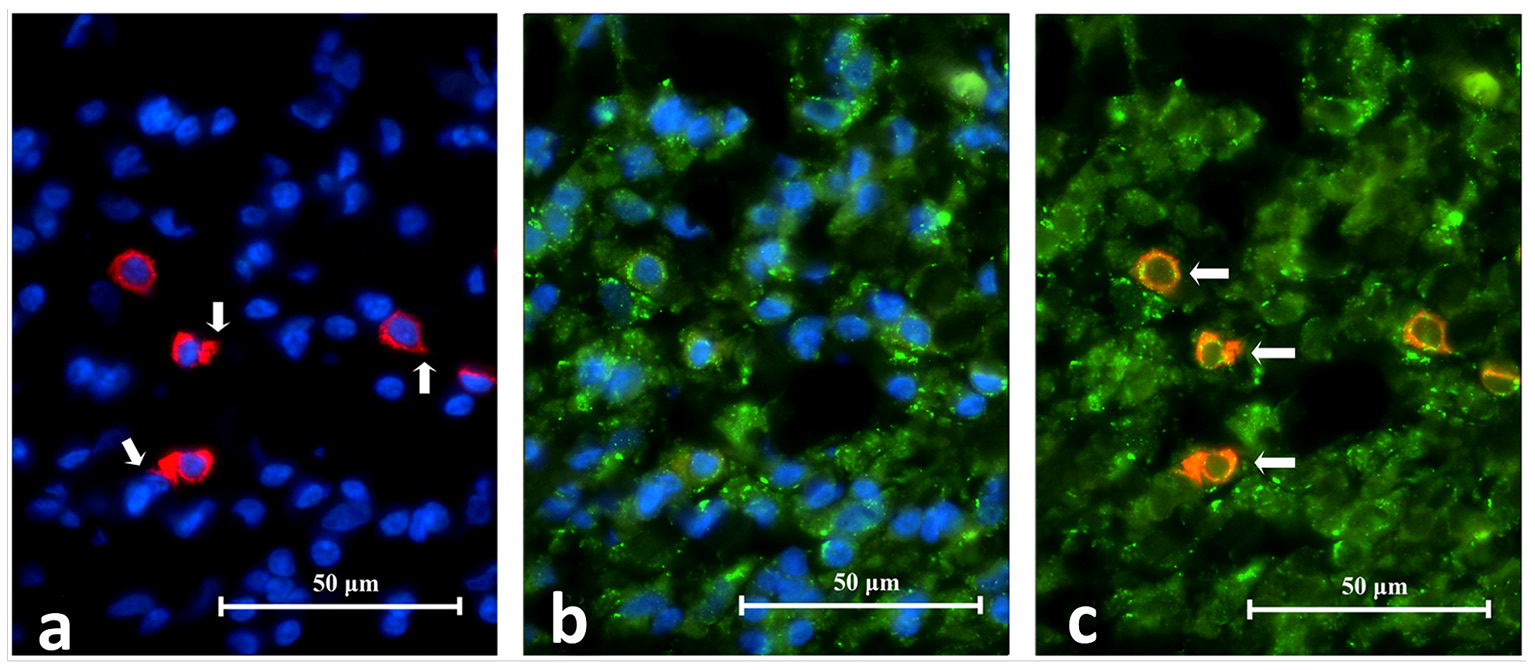
High power magnification images of an area in periapical granuloma captured under individual fluorescence. Image **A** shows CD83+ cells fluorescing in red. Notice the cell membrane, cytoplasm and cytoplasmic extension (white arrows) are stained. Image **B** shows TLR2+ cells fluorescing in green. The superimposed image **C** reveals CD83+/TLR2+ cells (white arrows) which have cell membrane and cytoplasm fluorescing in orange or a mixture of red and green speckles (CD68/TLR2 Double Immunofluorescence x1000).

The co-expression of CD83 and TLR2 were identified in six periapical granuloma samples. Under high power magnification both CD83 and TLR2 fluorescence were observed on the cell membrane and cytoplasm. The CD83 ^**+**^/TLR2 ^**+**^ cells fluoresced as a mixture of orange, red and green lights ( Figure 12). These cells exhibited thorn-like extensions and resembled dendritic cells in the lingual tonsil and were sparingly distributed throughout a lesion. The results are consistent with dendritic cell expression of TLR2 in periapical granuloma ( Figure 13).

Microscopic images for immunohistochemistry, immunofluorescence and double immunofluorescence TLR2 expression in different inflammatory tissues Click here for additional data file. Copyright: © 2018 Chen E et al. 2018 Data associated with the article are available under the terms of the Creative Commons Zero "No rights reserved" data waiver (CC0 1.0 Public domain dedication).

## Discussion

In a long-standing apical periodontitis, a periapical granuloma is the most common pathological entity ^33, 34^. The lesion itself is a granulomatous tissue infiltrated with inflammatory cells and encapsulated by a fibrous capsule. Lymphocytes are the dominant inflammatory cell in the lesion followed by macrophages, plasma cells, and neutrophils. The capsule mainly consists of collagen fibres and fibroblasts. Blood vessels and sometimes odontogenic epithelial cells are found interspersed throughout the entire lesion ^35^. This is consistent with the histological picture observed in the current study. Neither acute inflammatory cells nor areas of necrosis were evident in the selected cases within the present study as they were all refractory periapical granuloma cases with no signs of acute exacerbation. Lymphocytes being the most numerous cells in periapical granuloma, they showed the most abundant TLR ^+^ reactivity.

Residual microbes in the apical portion of the root canal system is a major cause of apical periodontitis persisting post-treatment in both poorly and well treated cases ^2^. In symptomatic refractory cases, the microbes found are those similar to primary endodontic infections ^36^. In well-treated endodontic cases the number of species that were recovered was typically between 1 to 5 species per canal ^37^ and was mostly Gram-positive facultative or obligatory anaerobes ^1^. A wide diversity of microbes had been isolated in different studies but *Enterococci* are the most reported species with prevalence between 32–77%, followed by *Streptococcus* (20–33%) and *Actinomyces* (3–27%) with *Candida*, a fungal species, making up a significant proportion (3–9%) ^20– 24, 33– 41^. TLR2 recognizes a number of components on cell walls of a variety of microorganisms including peptidoglycan and lipoteichoic acid (LTA) from Gram-positive bacteria ^13^. This explains the constant expression of TLR2 throughout all cases of refractory periapical granuloma. Lipoteichoic acids (LTA) of Gram-positive bacteria are one of the pathogen-associated molecular patterns (PAMPs) that are conserved, unchangeable and essential for the survival or pathogenicity of the microorganisms ^3^. Therefore, the presence of pattern recognition receptors (PRRs) including TLR2 is an essential mechanism of pathogen recognition in innate immunity ^4^.

The results showed a positive reaction to TLR2 by odontogenic epithelial cells within the refractory periapical granuloma cases. This is in agreement with a number of other studies where members of the TLR family were identified in a number of epithelial tissues within the body. Examples include epithelial cells on the mucosal surface of the intestinal tract having TLR5 expressed on their basolateral surface ^42^, and TLR4 expression at relatively low levels in intestinal epithelial cells, which may explain why LPS does not elicit a strong inflammatory response in the intestine ^43^. In contrast, intestinal epithelium from patients with inflammatory bowel diseases showed augmented expression of TLR4 ^44^.

TLR2 ligand recognition, unlike TLR4, involves cooperation with other TLR family members, particularly TLR6 and TLR1. Studies have shown that macrophages in mice that have competent TLR2 but are deficient in TLR6 failed to recognise microbial lipopeptides ^45, 46^. The same conclusion was made when a similar experiment was performed on TLR1-deficient mice ^47^. TLR6 and TLR1 are highly homologous and they may, in some cases, compensate for each other when one is deficient ^48^. It is unknown whether TLR2 forms a dimer with other TLRs constitutively or in response to ligand stimulation. This could explain the fact that TLR2 was not expressed in some of the immune cells within refractory periapical granuloma, as it might have been combined with other members of the TLR family within lesions. Furthermore, Expression of TLRs is modulated by a variety of factors such as microbial invasion, microbial components and cytokines. Colony-stimulating factor 1 ^7^, macrophage immigration inhibitory factor ^8^, interferon (INF) -γ ^9^, IL-2, IL-15, IL-1β and TNF-α ^10^ are examples of cytokines that prime phagocyte response to microbial stimulation, up- or down- regulate TLR gene expression and augment inflammatory cytokine production. Within refractory periapical granuloma, a large number of the above mentioned inflammatory cytokines are produced as a host defensive response and could contribute to the expression of TLR2 within the lesions.

The consistent expression of TLR2 within refractory periapical granuloma reported in the current study is in agreement with a number of other studies in the literature. Immunohistochemistry investigations on human refractory endodontic lesions only revealed TLR2 but failed to consistently demonstrate TLR4 expression ^23^. The consistent expression of TLR2 in the refractory periapical granuloma supports the observation that Gram-positive bacteria play a dominant role in the root canal infection of these lesions. Other studies investigated the expression of TLR2 in root resorption. Concomitant infection originating from the root canal or the periodontium may initiate an inflammatory response which resorbs unprotected dentine ^49, 50^. Lesions isolated from internal, external inflammatory root resorption as well as cervical root resorption have been identified to express TLR2 and TLR4 ^51^. Although the causative factors in each resorption may be different or somehow obscure, the expression of TLRs strongly suggests that bacteria play the central role of their pathogenesis.

Furthermore, the expression of TLR2 and TLR4 was investigated in the furcal periodontal ligament of the rat molar ^24^. Real-time PCR analysis of the furcal periodontal ligament showed that an unsealed pulpotomy exposed to the oral cavity increased the gene expression of TLR2 and TLR4, as well as several APC markers (MHC-II, CD83 and CD86 also known as T-Lymphocyte activation antigen) at 24 hours. Histochemical analysis also confirmed the presence of TLR2 and TLR4 positive cells in the inflamed periodontal ligament. Finally, TLR2 and TLR4 expressions in the dental pulp has been studied using a murine dental caries model. TLR2 and CD64 positive cells were found in abundance around the odontoblastic layer and at the centre of the pulp, while TLR4 positive cells were very few in both areas. Nonetheless, it was evident that both TLR2 and TLR4 were induced on the macrophages and dendritic-like cells at the early stage of pulpitis. However, it was reported that mRNA concentration for TLR2 was always higher than that of TLR4, reflecting the dominant role of Gram-positive bacteria in the early carious lesions ^19, 20^. The consensus from the previous studies provides solid evidence to support the findings from the present study that TLR2 plays a key role in the immune response against long standing bacterial infections, especially gram +ve predominant lesions.

Our results showed that neutrophils, RBCs, the collagen ground substance and the negative control samples from periapical scar tissues showed a negative reaction to immunostaining with TLR2 antibody. This is due to the absence of the stimuli needed for TLR2 activation such as bacterial antigens and inflammatory conditions as hypothesized by a previous study that investigated the expression of TLR2 in periapical lesions ^52^. The same study is in accordance with the current study in terms of TLR2 expression within macrophages with foamy appearance ^52^. The foamy appearance is linked to the production of nitric oxide and reactive oxygen species (ROS) necessary for the pathogenesis of chronic periapical lesions, rather than being a result of necrotic and/or apoptotic TLR2 ^+ ^cells ^53^. TLR2 might play a role in the production of ROS after activation by bacterial antigen, together with macrophages ^54^. Further studies are necessary to clarify the possible correlation between TLR2, macrophages and ROS in the pathogenesis of periapical lesions.

The characterization of TLR2 ^+^ cells requires the co-expression of TLR2 with an immune cell marker. It is important that the paired antibodies do not cross react during immunostaining and give a false identification. There are several methods to reduce the cross reactivity, all of which are based on the principle of sequential antibody application and the removal or destruction of the binding sites after each antigen-antibody interaction ^55, 56^. However, these methods are time consuming and greatly increase the chance of procedural errors. The most effective way is to use primary antibodies raised in different species ^57, 58^, allowing them to be pre-mixed in a single cocktail of antibody diluents and react with the antigens in one application. The same principle was also followed in the present study when choosing the labeling antibodies in IF.

In the present study, various immune cells present in refractory periapical granuloma were identified to express TLR2 which was in agreement with Desai *et al*. (2011) ^59^. The most common TLR2 ^+^ cells in refractory periapical granuloma resembled the morphology of lymphocytes and macrophages. Finally, the odontogenic epithelium found interspersed in the lesion also expressed TLR2 although this was not reported by Desai *et al*. (2011) ^59^. In contrast to Hayashi *et al*. (2003) ^60^ but in line with Desai *et al*. (2011) ^59^, the neutrophils identified in the lesions (characterised by their multi-lobed nucleus) did not reveal the presence of TLR2. The differences may be due to the methodology employed – Hayashi *et al*. (2003) ^60^ used isolated cells in flow cytometry analysis which is more sensitive in antigen detection than immunofluroscence on tissue sections ^61^.

In the current study, TLR2 stain was located on the cell surface as well as cytoplasm. Two reasons can be given to explain this observation: biologically, TLR2 is actively synthesized in the cytoplasm and thus it was stained. Technically, the histology section (which is 4 µm in thickness) contains a significant portion of a cell in three dimensions (typical lymphocytes are about 8 to 10 μm in diameter) and thus the stained cell membrane in three dimensions is viewed as stained cytoplasm.

CD38 is a cell surface enzyme involved in transmembrane signaling and cell adhesion ^62^. It is expressed in activated T cells, B cells and plasma cells ^63^. CD38 was selected for this study because it encompasses the entire range of lymphocytes. CD38 ^+^ cells representing lymphocytes and plasma cells dominated the infiltrated immune cell population in the refractory periapical granuloma which is consistent with most studies ^28, 31^. In areas of secondary abscess where macrophages, monocytes and neutrophils are found, no CD38 ^+^ cells could be identified.

CD68 is a transmembrane glycoprotein highly expressed by human monocytes and tissue macrophages ^64, 65^. In this study, monocytes and macrophages were identified in 5 samples. They were distinguished according to their morphology, macrophages are large cells (up to 25 µm across) with foamy cytoplasm and monocytes are smaller cells (about 10 µm) with a circular profile. They were found abundantly in areas of secondary abscess which also revealed numerous neutrophils (identified by multi-lobed nucleus). No CD68 ^+^ cells were found outside an area of abscess. Although not quantitatively analyzed, the number of macrophages and monocytes were relatively high which was consistent with Stern *et al*. (1981) ^25^, Johannessen *et al*. (1984) ^66^, Nilsen *et al*. (1984) ^27^, Kopp & Schwarting (1989) ^29^, and Piattelli *et al*. (1991) ^30^ but not Bergenholtz *et al*. (1983) ^26^ or Kontiainen *et al*. (1986) ^28^. The proportion of macrophages in the apical lesion has been suggested to correlate positively with clinical signs and symptoms ^67^. All samples selected in the current study were symptomatic which may account for the substantial number of macrophages observed.

Three samples failed to show CD68 ^+^ immunofluroscent cells. Two reasons may be given for their absence. Firstly, periapical granuloma are heterogenous in cellular and structural components ^35^. The composition of cells residing in the periapical granuloma can shift in response to the dynamic relationship between the microbial and host factors. In addition, it has been shown that a secondary abscess, where macrophages and neutrophils were found in the present study, can occur anywhere inside the granulation tissue and not necessarily close to the apical foramen ^35^. Therefore, the level where the tissue is sectioned may not include the macrophage-rich areas. Secondly, the tissue samples stored in the archives do not represent the entire lesion because a significant portion had already been removed during the routine diagnostic procedure. Furthermore, the lesions may not always be enucleated completely at the surgery. Some tissue portions may have been left in the bony crypt, lost through the suction tip or were removed in several pieces.

CD83 is a glycoprotein that is expressed on the cell membrane of mature dendritic cells (DC) ^68, 69^. In this study, six samples had identified CD83 ^+^ cells which exhibited variable body shape (about 10 µm across) and many showed small to large cytoplasmic extensions that are characteristic of dendritic cells ^32^. Although not quantitatively analyzed, the CD83 ^+^ cells were infrequently observed and randomly distributed in the lesion. This was consistent with Liapatas *et al*. (2003) ^31^ and Lukić *et al*. (2006) ^32^ who quantitatively showed DCs have weak expression (0-10 % and 0.4–1.2% of the total infiltrated cell population respectively) in periapical lesions.

Desai *et al*. (2011) ^59^ was the first group to characterize TLR2 ^+^ cells in periapical lesions using serial section staining by methyl green pyronin (plasma cells) and immunoperoxidase (CD3 and CD19 cells) techniques. Although the expression of TLR2 on T cells (CD3 ^+^), B cells (CD19 ^+^), plasma cells and macrophages were revealed, the authors acknowledged that their identification was not definitive and more specific cell marking was required to determine cell phenotypes. In this study the DIF technique was employed to overcome the lack of specificity in co-localizing cell markers using serial staining. Both the TLR2 and the respective CD markers were located on the cell membrane and cytoplasm although the intensities and patterns of the fluorescence varied. Overall, the study was in agreement with Desai *et al*. (2011) ^59^. Furthermore, the current study has successfully marked monocytes (CD68 ^+^) and mature dendritic cells (CD83 ^+^) and supports the speculation that TLR2 ^+^CD3 ^-^CD19 ^-^ MGP cells could be dendritic cell subsets or macrophages ^59^.

In this study, mature DCs were observed scattered throughout the lymphocyte rich areas and some appeared physically close to them. Since mature DCs are highly mobile units, this may suggest they are actively moving and performing antigen presentation to local lymphocytes but the minute number of mature DC observed remain unexplained. However, the population of DCs in all organs is maintained through a dynamic balance of three parameters: continuous input of pre-DCs from blood, limited DC division *in situ*, and cell death ^70^. Since mature DCs ultimately migrate to the local lymph nodes, their small presence in periapical granuloma may be due to the difference between their exodus and replenishment from circulating pre-DCs.

Toll-like receptor 2 expressed on B cells may play two roles within the periapical granuloma. Firstly, it may work with the surface immunoglobulin in taking up the antigen thus facilitating B cells as Antigen Presenting Cells (APC). Secondly, the ligation of LTA to TLR2 on memory B cells may reactivate B cell immune responses without signals from T cells ^71, 72^. In contrast to memory B cells, the expression of TLRs on plasma cells remains unknown in the current literature. Nonetheless, the possibility that plasma cells express TLR2 has been suggested ^59, 72^ and appeared likely in this study. It has been hypothesized that direct activation of plasma cells by TLR ligands could contribute to plasma cell-derived IgG secretion ^72^.

In this study, macrophages were identified to express TLR2. Unlike dendritic cells, macrophages were abundant but only located in the areas of secondary abscesses and they were not found scattered throughout the lymphocyte-rich regions. This may suggest the main role of macrophages in the refractory periapical granuloma is to recognize microbes through TLR2 and to perform phagocytosis. Antigen presentation to lymphocytes may be a lesser role for macrophages and only occurs to nearby lymphocytes. In this study, macrophages expressed TLR2 both on the cell membrane and cytoplasm. Since the size of a macrophage is typically 20 µm, the detection of the TLR2 in the cytoplasm is best explained by the role they play in periapical granuloma. To sustain effective microbe recognition and their removal, the cell membrane of the macrophage requires constant resupply of TLR2 and thus TLR2 protein synthesis is up-regulated in the cytoplasm. Furthermore, as a scavenger, the positive staining may represent the phagocytosed cellular debris that had expressed TLR2 ^59^.

Further investigations to identify lymphocytes and their sub-populations have yet to be disclosed. The identification of these lymphocytes and their expression of TLR2 may provide an insight into the effect these cells have on the pathogenesis of refractory periapical granuloma. Furthermore, a quantitative analysis based on flow cytometry could be supplemented to obtain additional information on the proportion and intensity of TLR2 expression amongst immune cells. Based on this research model, periapical lesions of endodontic origin with varied characteristics (such as primary or secondary, symptomatic or asymptomatic, granuloma or cyst) could be explored with different classes of TLR. Toll-like receptor pathways act as a bridge between the innate and adaptive immunity. Detailed investigations on the pattern of TLRs expression and cellular interaction may contribute new discoveries regarding the dynamic relationship between bacteria and host, the development and growth of the lesion and future treatment strategies.

## Conclusion

The protocol for immunohistochemical procedures of TLR2, CD38, CD68 and CD83 have been standardized and the expression of each cell marker (TLR2, CD38, CD68 and CD83) within refractory periapical granuloma was identified. Refractory periapical granuloma consistently expressed TLR2 through lymphocytes and plasma cells (CD38 ^**+**^), macrophages and monocytes (CD68 ^**+**^) and mature dendritic cells (CD83 ^**+**^). Lymphocytes and plasma cells appeared to be the dominant inflammatory cells expressing TLR2. Although CD68 ^**+**^ and CD83 ^**+**^ cells may not always be present in a lesion, they did express TLR2 whenever they were identified. TLR2 ^+^ dendritic cells play a minor role in periapical granuloma with regards to antigen recognition. The current study confirms previous findings that highlight the significant role of Gram-positive bacteria in clinical cases of refractory periapical granuloma. This was demonstrated through the expression of TLR2 in symptomatic cases.

## Data availability

The data referenced by this article are under copyright with the following copyright statement: Copyright: © 2018 Chen E et al.

Data associated with the article are available under the terms of the Creative Commons Zero "No rights reserved" data waiver (CC0 1.0 Public domain dedication). 

All microscopic images are available in the following dataset including microscopic images for positive control and negative control cases. The zip file contains a key for the microscope image files.

F1000Research: Dataset 1. Microscopic images for immunohistochemistry, immunofluorescence and double immunofluorescence TLR2 expression in different inflammatory tissues, https://dx.doi.org/10.5256/f1000research.16678.d224146 ^73^
