# Supplementary material for: Inflammatory cell expression of Toll-like receptor-2 (TLR2) within refractory periapical granuloma
Source: F1000Res. 2018 Nov 20;7:1819. [Version 1] doi: 10.12688/f1000research.16678.1 (PMC6281009; doi:10.12688/f1000research.16678.1)
Supplement: Peer review history

# Peer review: Referee response for version 1

**DOI:** 10.5256/f1000research.18229.r40929

**Published:** 2018-11-30

**Authors:** Lakshmi Thangavelu, Aravind Kumar.S

**Affiliations:** 1 Department of Pharmacology, Saveetha Dental College And Hospitals, Saveetha Institute of Medical and Technical Sciences (SIMATS), Chennai, Tamil Nadu, India; 2 Department of Orthodontics, Saveetha Institute of Medical and Technical Sciences, Chennai, Tamil Nadu, India

In this manuscript Bakr *et al.* gave promising evidence about the protocol employed in immunohistochemical analysis of various immunomarkers. This research provides brief information about the role of Toll-like receptors and their applications in clinical practice for diagnosis. I was curious to know about the sample size selected in the study - when I went through the criteria for selecting the sample size was found to be satisfactory. The introduction part of the article provides lots of information about the Toll-like receptors’ role in the field of endodontics. The authors highlighted the immunohistochemical analysis well in the manuscript.

 Minor comments: 

1. The introduction is too elaborate. I would appreciate it if the key points are focused on.
2. Even though the sample size utilised is 8 for qualitative analysis out of 772 samples, this needs further explanation.
3. The statistical analysis is not incorporated in the study to show the significance.

Overall the authors have done excellent work. The authors have provided a brief insight about the Toll-like receptors and refractory periapical lesions.

 If the points raised above will be addressed, indexing is appropriate for this manuscript.

We have read this submission. We believe that we have an appropriate level of expertise to confirm that it is of an acceptable scientific standard.

# Peer review: Referee response for version 1

**DOI:** 10.5256/f1000research.18229.r40928

**Published:** 2018-11-26

**Authors:** Paul Vincent Abbott

**Affiliations:** 1 UWA Dental School, University of Western Australia, Nedlands, WA, Australia

This paper has addressed a particular clinical problem that is often found in specialist Endodontic practices - i.e. refractory cases that do not heal and show persistent radiolucency despite all the best clinical endodontic procedures and various anti-bacterial strategies being used. The authors have investigated the role of Toll-like receptor-2 (TLR-2) in these refractory periapical lesions. They showed that lymphocytes and plasma cells from these lesions have high expression levels of TLR-2 which indicates their role in these cases. This information has the potential to lead to better diagnostic and management strategies to identify these refractory cases earlier.

 This is a well-conducted study that has utilized samples of refractory cases from the university’s collection of cases. The methodology involved numerous tests and analysis of the biopsy material along with positive and negative controls to support the methodology used. The paper has comprehensive descriptions and histological material to support the discussion and conclusions. The results support previous studies.

I have read this submission. I believe that I have an appropriate level of expertise to confirm that it is of an acceptable scientific standard.
